# Supplementary figures and images for: The use of spatial data and satellite information in legal compliance and planning in forest management
Source: PLoS One. 2022 Jul 27;17(7):e0267959. doi: 10.1371/journal.pone.0267959 (PMC9328540; doi:10.1371/journal.pone.0267959)

**Figure S1. QQ Plots assessing for normalcy for Elevation and calculated Slope data**


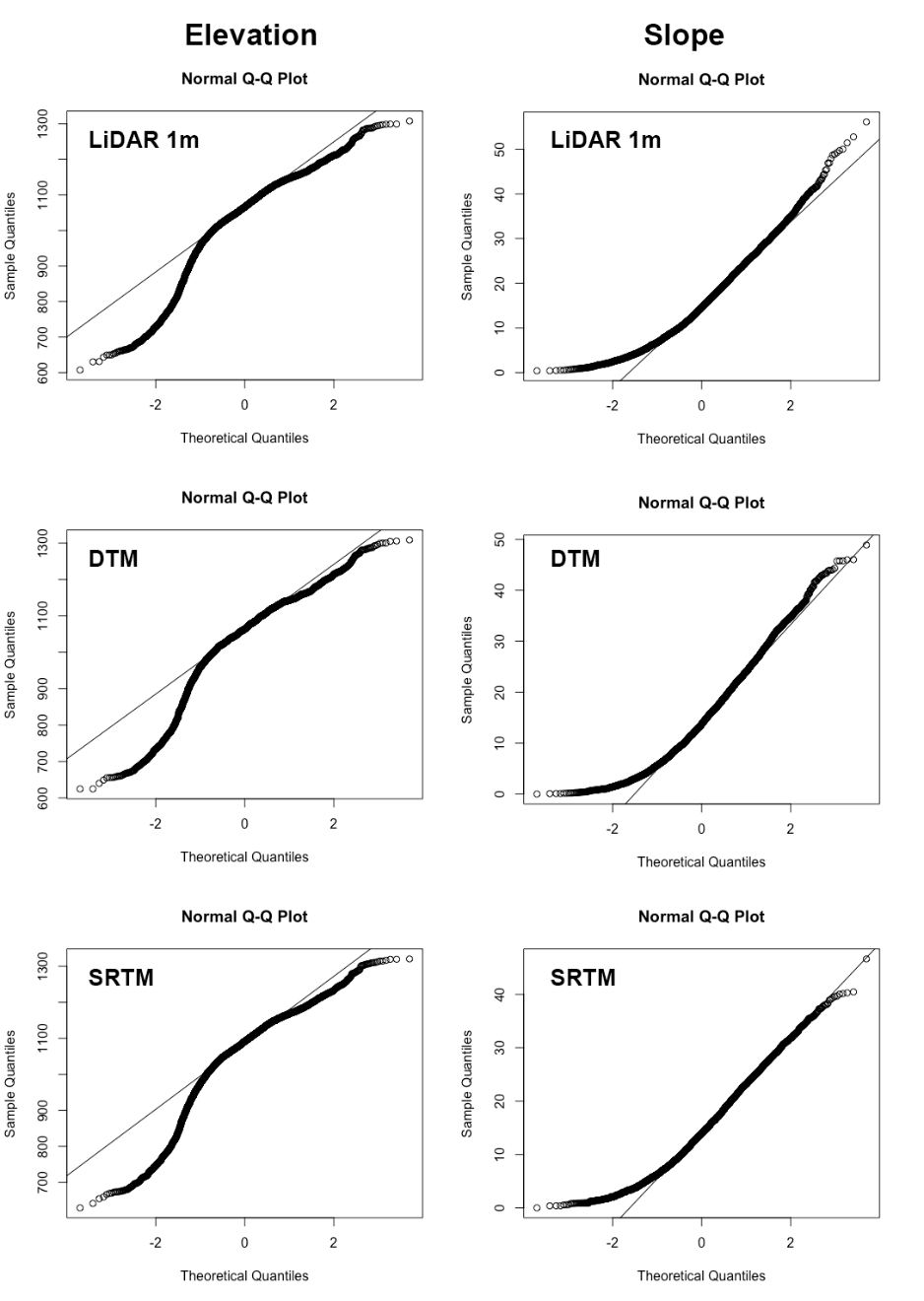

Supplement: S1 Fig — (DOCX) [file pone.0267959.s001.docx]

**Figure S2. QQ Plots assessing for normalcy for Cut Block Area >30° in Slope**

**
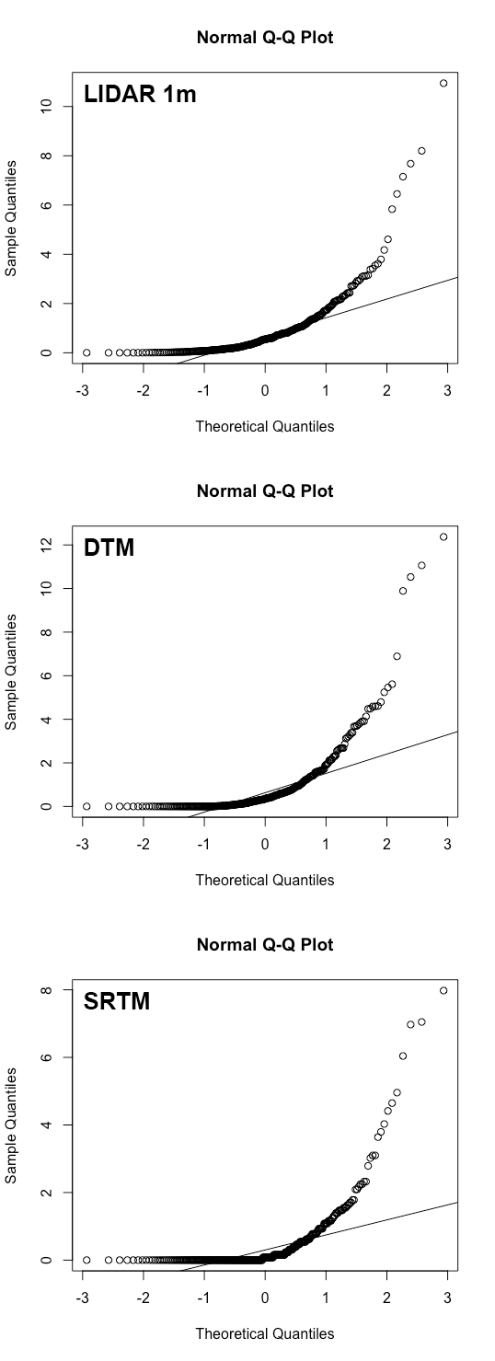
**

Supplement: S2 Fig — (DOCX) [file pone.0267959.s002.docx]
