# Supplementary material for: The use of spatial data and satellite information in legal compliance and planning in forest management
Source: PLoS One. 2022 Jul 27;17(7):e0267959. doi: 10.1371/journal.pone.0267959 (PMC9328540; doi:10.1371/journal.pone.0267959)
Supplement: S3 Fig — LiDAR derived slope at 1m resolution (S3A Fig); LiDAR derived slope with an average slope neighbourhood radius of 5m (S3B Fig); STRM derived slope at 1 arc second resolution (S3C Fig); DTM derived slope at 10m resolution (S3D Fig). (DOCX) [file pone.0267959.s003.docx]

**Figure S3. Comparison slope calculations with transects measured on cut block 318-512-0018 by the Authors. LiDAR derived slope at 1m resolution (Figure S3A); LiDAR derived slope with an average slope neighbourhood radius of 5m (Figure S3B); STRM derived slope at 1 arc second resolution (Figure S3C); DTM derived slope at 10m resolution (Figure S3D).**

**
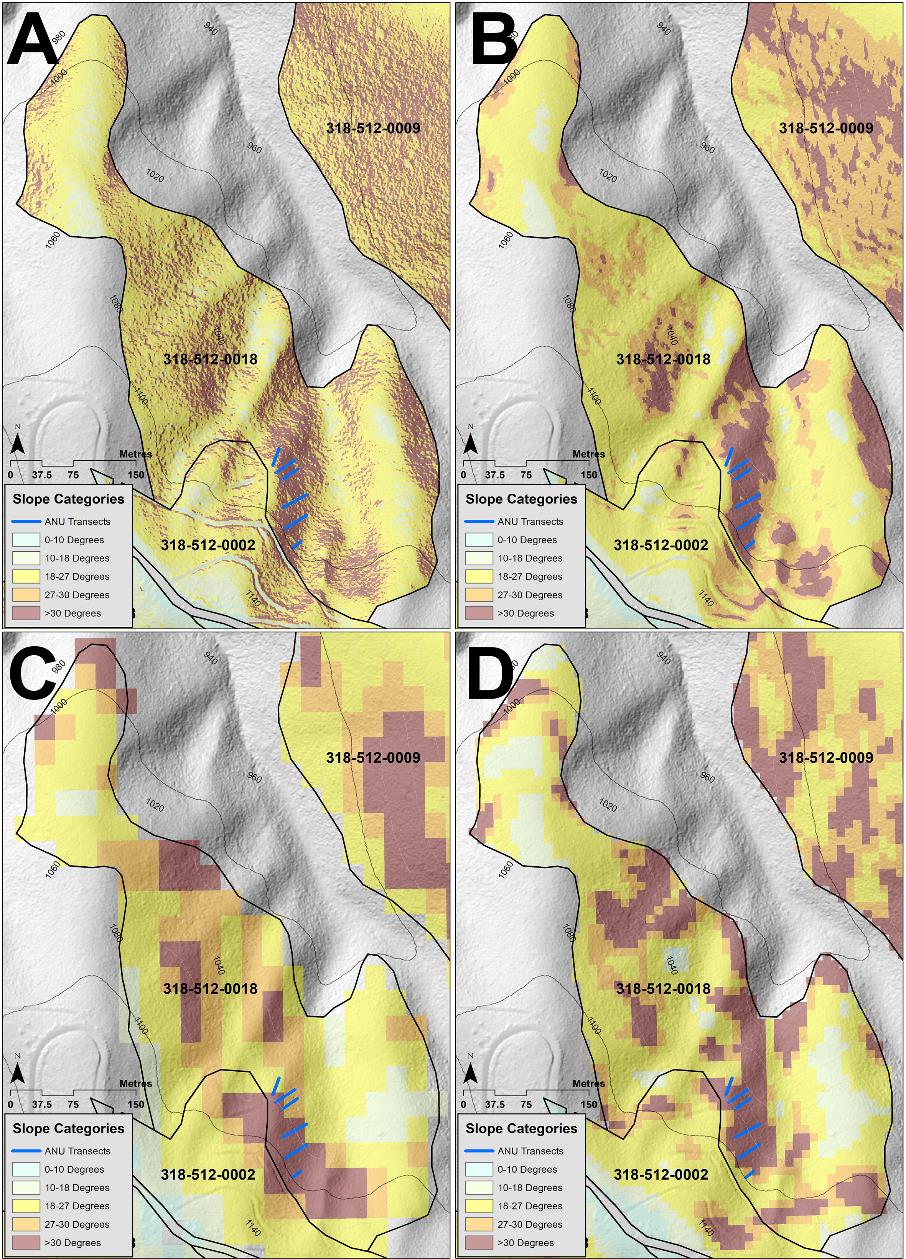
**
