# Supplementary material for: The use of spatial data and satellite information in legal compliance and planning in forest management
Source: PLoS One. 2022 Jul 27;17(7):e0267959. doi: 10.1371/journal.pone.0267959 (PMC9328540; doi:10.1371/journal.pone.0267959)
Supplement: S4 Fig — LiDAR derived slope at 1m resolution (S4A Fig); LiDAR derived slope with an average slope neighbourhood radius of 5m (S4B Fig); STRM derived slope at 1 arc second resolution (S4C Fig); DTM derived slope at 10m resolution (S4D Fig). (DOCX) [file pone.0267959.s004.docx]

**Figure S4. Comparison slope calculations with transects measured on cut block 457-504-0006 for this study. LiDAR derived slope at 1m resolution (Figure S4A); LiDAR derived slope with an average slope neighbourhood radius of 5m (Figure S4B); STRM derived slope at 1 arc second resolution (Figure S4C); DTM derived slope at 10m resolution (Figure S4D).**

**
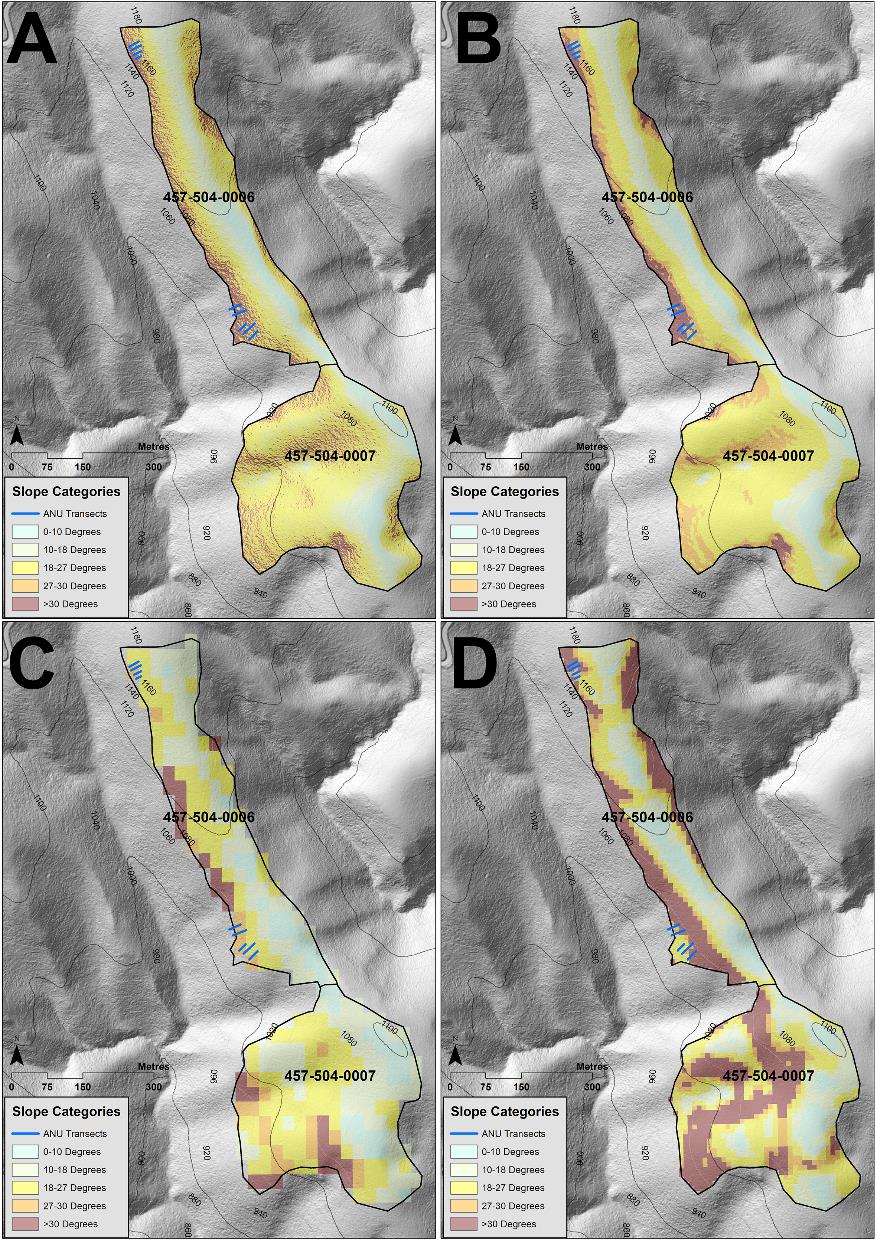
**
