# Supplementary material for: The use of spatial data and satellite information in legal compliance and planning in forest management
Source: PLoS One. 2022 Jul 27;17(7):e0267959. doi: 10.1371/journal.pone.0267959 (PMC9328540; doi:10.1371/journal.pone.0267959)

**Figure S5. QQ Plots assessing for normalcy across Transects measured for this study (ANU Transects) and by the Timber Harvest Compliance Unit of the OCR (OCR Transects).**


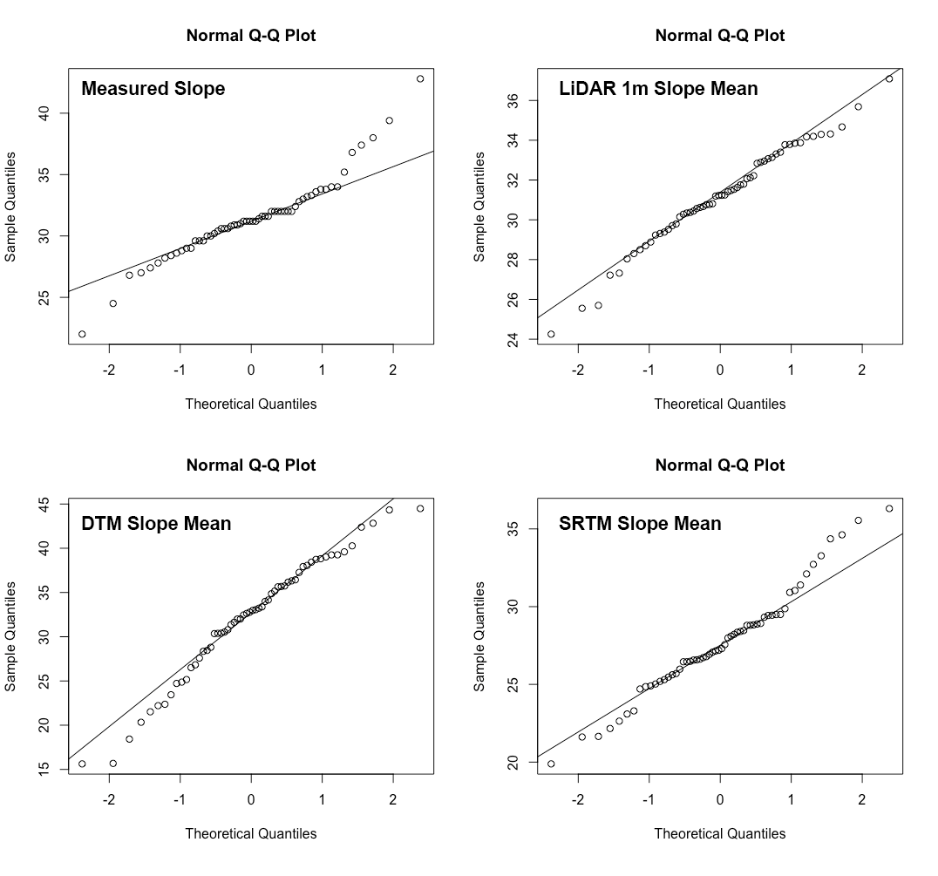

Supplement: S5 Fig — (DOCX) [file pone.0267959.s005.docx]
