# Supplementary material for: The use of spatial data and satellite information in legal compliance and planning in forest management
Source: PLoS One. 2022 Jul 27;17(7):e0267959. doi: 10.1371/journal.pone.0267959 (PMC9328540; doi:10.1371/journal.pone.0267959)
Supplement: S1 Table — (DOCX) [file pone.0267959.s006.docx]

**Table S1. Moran’s I Spatial Autocorrelation for the analysis grid points across cut blocks in the Upper Goulburn and Thomson water supply projection areas**

| Type | Data | Moran's Index | Expected Index | Variance | Z-Score | P-Value | Category |
| --- | --- | --- | --- | --- | --- | --- | --- |
| Slope | LiDAR 1m | -0.056 | -0.015 | 0.009 | -0.429 | 0.668 | Random |
|  | LiDAR F5m | -0.088 | -0.015 | 0.009 | -0.763 | 0.446 | Random |
|  | DTM | 0.037 | -0.015 | 0.009 | 0.542 | 0.588 | Random |
|  | SRTM | 0.052 | -0.015 | 0.009 | 0.710 | 0.478 | Random |
| Elevation | LiDAR 1m | 0.739 | -0.015 | 0.009 | 7.894 | 0.000 | Clustered |
|  | DTM | 0.720 | -0.015 | 0.009 | 7.689 | 0.000 | Clustered |
|  | SRTM | 0.744 | -0.015 | 0.009 | 7.940 | 0.000 | Clustered |
