# Supplementary material for: The use of spatial data and satellite information in legal compliance and planning in forest management
Source: PLoS One. 2022 Jul 27;17(7):e0267959. doi: 10.1371/journal.pone.0267959 (PMC9328540; doi:10.1371/journal.pone.0267959)
Supplement: S2 Table — (DOCX) [file pone.0267959.s007.docx]

**Table S2. Test for Normalcy using the Shapiro-Wilks Test and QQPlots for the Elevation, Slope, Cut Block Area >30° and the Transects measured on site (ANU and OCR)**

| Component | Data | W Value | p-value | Shapiro-Wilks Test | QQ Plot |
| --- | --- | --- | --- | --- | --- |
| Elevation | LiDAR 1m Elevation | 0.91 | < 2.2e-16 | Non-Parametric | Non-Parametric |
|  | DTM Elevation | 0.92 | < 2.2e-16 | Non-Parametric | Non-Parametric |
|  | SRTM Elevation | 0.91 | < 2.2e-16 | Non-Parametric | Non-Parametric |
| Slope | LiDAR 1m Slope | 0.97 | < 2.2e-16 | Non-Parametric | Parametric |
|  | LiDAR F5m Slope | 0.98 | < 2.2e-16 | Non-Parametric | Parametric |
|  | DTM Slope | 0.97 | < 2.2e-16 | Non-Parametric | Parametric |
|  | SRTM Slope | 0.98 | < 2.2e-16 | Non-Parametric | Parametric |
| Cut Block Area >30° | LiDAR 1m | 0.63 | < 2.2e-16 | Non-Parametric | Non-Parametric |
|  | LiDAR F5m | 0.44 | < 2.2e-16 | Non-Parametric | Non-Parametric |
|  | DTM | 0.60 | < 2.2e-16 | Non-Parametric | Non-Parametric |
|  | SRTM | 0.52 | < 2.2e-16 | Non-Parametric | Non-Parametric |
| ANU Transects | Slope Measured | 0.89 | 0.008 | Non-Parametric | Non-Parametric |
|  | LiDAR 1m | 0.94 | 0.134 | Parametric | Parametric |
|  | DTM | 0.93 | 0.083 | Parametric | Parametric |
|  | SRTM | 0.956 | 0.319 | Parametric | Parametric |
| OCR Transects | Slope Measured | 0.95 | 0.131 | Parametric | Parametric |
|  | LiDAR 1m | 0.93 | 0.042 | Non-Parametric | Non-Parametric |
|  | DTM | 0.93 | 0.024 | Non-Parametric | Non-Parametric |
|  | SRTM | 0.97 | 0.559 | Parametric | Parametric |
