# Supplementary material for: The use of spatial data and satellite information in legal compliance and planning in forest management
Source: PLoS One. 2022 Jul 27;17(7):e0267959. doi: 10.1371/journal.pone.0267959 (PMC9328540; doi:10.1371/journal.pone.0267959)
Supplement: S3 Table — (DOCX) [file pone.0267959.s008.docx]

**Table S3. Descriptive Statistics for the Elevation (mASL) and difference between the LiDAR 1m DEM and the VicMap Elevation DTM and SRTM DEM (m)**

|  | DEM | Min | 1st Qu | Median | Mean | 3rd Qu | Max | Sd |
| --- | --- | --- | --- | --- | --- | --- | --- | --- |
| Elevation (mASL) | LiDAR | 607.5 | 1004.6 | 1065.9 | 1047.2 | 1128.1 | 1307.8 | 114.46 |
|  | DTM | 624.7 | 1003.4 | 1063.9 | 1046.4 | 1123.8 | 1308.6 | 113.03 |
|  | SRTM | 629.3 | 1025.4 | 1091.9 | 1069.5 | 1149.8 | 1320.3 | 116.18 |
| Difference (m) | LiDAR-DTM | -52.63 | -4.5599 | 1.44 | 0.8867 | 7.075 | 56.362 | 10.45 |
|  | LiDAR-SRTM | -48.97 | -27.98 | -22.29 | -22.27 | -16.68 | 18.16 | 8.64 |
