# Supplementary material for: The use of spatial data and satellite information in legal compliance and planning in forest management
Source: PLoS One. 2022 Jul 27;17(7):e0267959. doi: 10.1371/journal.pone.0267959 (PMC9328540; doi:10.1371/journal.pone.0267959)
Supplement: S4 Table — (DOCX) [file pone.0267959.s009.docx]

**Table S4. Moran’s I Spatial Autocorrelation for the elevation difference between LiDAR analysis grid points and the VicMap Elevation DTM and SRTM DEM across cut blocks in the Upper Goulburn and Thomson water supply projection areas**

| Type | Data | Moran's Index | Expected Index | Variance | Z-Score | P-Value | Category |
| --- | --- | --- | --- | --- | --- | --- | --- |
| Elevation | DTM-LiDAR 1m | 0.363 | -0.0002 | 0.0006 | 46.535 | 0.000 | Clustered |
|  | SRTM- LiDAR 1m | 0.288 | -0.0002 | 0.0006 | 37.003 | 0.000 | Clustered |
