# Supplementary material for: The use of spatial data and satellite information in legal compliance and planning in forest management
Source: PLoS One. 2022 Jul 27;17(7):e0267959. doi: 10.1371/journal.pone.0267959 (PMC9328540; doi:10.1371/journal.pone.0267959)
Supplement: S5 Table — (DOCX) [file pone.0267959.s010.docx]

**Table S5. Descriptive Statistics for the Slope (mASL) and difference between the LiDAR 1m DEM and the VicMap Elevation DTM and SRTM DEM (m)**

|  | DEM | Min | 1st Qu | Median | Mean | 3rd Qu | Max | Sd |
| --- | --- | --- | --- | --- | --- | --- | --- | --- |
| Value | LiDAR 1m | 0.40 | 8.91 | 14.65 | 15.65 | 21.42 | 56.12 | 8.67 |
|  | LiDAR F5m | 1.18 | 9.79 | 15.12 | 15.70 | 21.00 | 38.25 | 7.37 |
|  | DTM | 0.00 | 7.90 | 13.72 | 14.85 | 20.71 | 48.89 | 8.89 |
|  | SRTM | 0.02 | 8.32 | 13.99 | 14.70 | 20.21 | 46.65 | 7.94 |
| Difference | LiDAR 1m-DTM | -34.94 | -3.97 | 0.86 | 0.80 | 5.65 | 40.11 | 8.25 |
|  | LiDAR 1m -SRTM | -38.33 | -3.80 | 0.65 | 0.95 | 5.37 | 42.15 | 7.62 |
|  | LiDAR F5m-DTM | -29.92 | -3.02 | 1.16 | 0.85 | 5.03 | 27.40 | 6.82 |
|  | LiDAR F5m -SRTM | -27.11 | -2.63 | 0.94 | 1.00 | 4.58 | 26.73 | 5.91 |
