# Supplementary material for: The use of spatial data and satellite information in legal compliance and planning in forest management
Source: PLoS One. 2022 Jul 27;17(7):e0267959. doi: 10.1371/journal.pone.0267959 (PMC9328540; doi:10.1371/journal.pone.0267959)
Supplement: S6 Table — (DOCX) [file pone.0267959.s011.docx]

**Table S6. Ordinary Least Squares test between the slope rasters generated from the LiDAR 1m DEM, including the LiDAR 1m and LiDAR F5m slope rasters, and the VicMap Elevation DTM and SRTM DEM**

|  |  | Estimate | Std Error | T Value | Pr(>ltl) | Significance |
| --- | --- | --- | --- | --- | --- | --- |
| LiDAR 1m - DTM | Intercept | 5.88 | 0.22 | 26.29 | <2e-16 | *** |
| LiDAR 1m - DTM | Slope | 0.57 | 0.01 | 45.82 | <2e-16 | *** |
| LiDAR 1m - SRTM | Intercept | 6.36 | 0.20 | 32.5 | <2e-16 | *** |
| LiDAR 1m - SRTM | Slope | 0.53 | 0.01 | 48.7 | <2e-16 | *** |
| LiDAR F5m - DTM | Intercept | 2.31 | 0.23 | 10.04 | <2e-16 | *** |
| LiDAR F5m - DTM | Slope | 0.80 | 0.01 | 60.10 | <2e-16 | *** |
| LiDAR F5m - SRTM | Intercept | 6.09 | 0.16 | 37.58 | <2e-16 | *** |
| LiDAR F5m - SRTM | Slope | 0.65 | 0.01 | 67.41 | <2e-16 | *** |
| Residuals | Min | 1Q | Median | 3Q | Max |  |
| LiDAR 1m - DTM | -25.14 | -5.24 | -0.80 | 4.34 | 32.37 |  |
| LiDAR 1m - SRTM | -25.17 | -4.45 | -0.47 | 4.13 | 35.86 |  |
| LiDAR F5m - DTM | -23.81 | -4.40 | -0.66 | 3.75 | 30.83 |  |
| LiDAR F5m - SRTM | -22.21 | -3.65 | -0.25 | 3.46 | 24.91 |  |
|  | Multiple R-squared | Adjusted R-squared | F-statistic | | Residual standard error | |
| LiDAR 1m - DTM | 0.31 | 0.31 | 2101 on 1 and 4624 DF | | 7.37 on 4624 degrees of freedom | |
| LiDAR 1m - SRTM | 0.34 | 0.34 | 2372on 1 and 4624 DF | | 6.45 on 4624 degrees of freedom | |
| LiDAR F5m - DTM | 0.44 | 0.44 | 3612 on 1 and 4624 DF | | 6.66 on 4624 degrees of freedom | |
| LiDAR F5m - SRTM | 0.50 | 0.50 | 4541 on 1 and 4625 DF | | 5.64 on 4624 degrees of freedom | |
