# Supplementary material for: The use of spatial data and satellite information in legal compliance and planning in forest management
Source: PLoS One. 2022 Jul 27;17(7):e0267959. doi: 10.1371/journal.pone.0267959 (PMC9328540; doi:10.1371/journal.pone.0267959)
Supplement: S8 Table — (DOCX) [file pone.0267959.s013.docx]

**Table S8. Descriptive Statistics for the area >30° logged in each cut block and difference between the LiDAR 1m DEM and the VicMap Elevation DTM and SRTM DEM (ha)**

|  | DEM | Min | 1st Qu | Median | Mean | 3rd Qu | Max | Sd |
| --- | --- | --- | --- | --- | --- | --- | --- | --- |
| Value | LiDAR 1m | 0.00 | 0.13 | 0.44 | 0.85 | 1.03 | 10.95 | 1.24 |
|  | LiDAR F5m | 0.00 | 0.00 | 0.05 | 0.35 | 0.35 | 8.14 | 0.82 |
|  | DTM | 0.00 | 0.02 | 0.29 | 0.90 | 1.11 | 12.33 | 1.56 |
|  | SRTM | 0.00 | 0.00 | 0.00 | 0.47 | 0.46 | 7.59 | 1.02 |
| Difference | DTM- LiDAR 1m | -2.23 | -0.32 | -0.05 | 0.05 | 0.27 | 4.67 | 0.88 |
|  | SRTM-LiDAR 1m | -5.14 | -0.63 | -0.25 | -0.37 | -0.05 | 3.97 | 0.74 |
|  | DTM- LiDAR F5m | -0.90 | 0.00 | 0.13 | 0.56 | 0.73 | 6.40 | 1.00 |
|  | SRTM- LiDAR F5m | -2.59 | -0.03 | 0.00 | 0.13 | 0.13 | 5.39 | 0.66 |
