# Supplementary material for: The use of spatial data and satellite information in legal compliance and planning in forest management
Source: PLoS One. 2022 Jul 27;17(7):e0267959. doi: 10.1371/journal.pone.0267959 (PMC9328540; doi:10.1371/journal.pone.0267959)
Supplement: S9 Table — (DOCX) [file pone.0267959.s014.docx]

**Table S9. Wilcoxon signed rank test comparing areas >30° in slope logged calculated by the LiDAR 1m, LiDAR F5m, VicMap Elevation DTM and the SRTM DEM**

| **Comparison** | **V** | **P-Value** |
| --- | --- | --- |
| LiDAR 1m and DTM | 28373 | 0.1561 |
| LiDAR 1m and SRTM | 45142 | < 2.2e-16 |
| LiDAR F5m and DTM | 5160.5 | < 2.2e-16 |
| LiDAR F5m and SRTM | 15887 | 0.04916 |
