# Supplementary material for: The use of spatial data and satellite information in legal compliance and planning in forest management
Source: PLoS One. 2022 Jul 27;17(7):e0267959. doi: 10.1371/journal.pone.0267959 (PMC9328540; doi:10.1371/journal.pone.0267959)
Supplement: S10 Table — (DOCX) [file pone.0267959.s015.docx]

**Table S10. ANOVA and Tukey’s HSD Test between Block 318 and other forest management blocks**

| Slope Raster | FMB Comparison | diff | lwr | upr | p adj |
| --- | --- | --- | --- | --- | --- |
| LiDAR 1m | 318-289 | 4.49 | 0.39 | 8.59 | 0.02 |
|  | 318-290 | 2.69 | -0.18 | 5.56 | 0.09 |
|  | 318-312 | 5.06 | 3.16 | 6.97 | 0.00 |
|  | 318-313 | 4.68 | 0.11 | 9.24 | 0.04 |
|  | 318-314 | 3.90 | 0.30 | 7.49 | 0.02 |
|  | 318-317 | 7.30 | 5.21 | 9.38 | 0.00 |
|  | 319-318 | -5.39 | -8.83 | -1.96 | 0.00 |
|  | 320-318 | -7.70 | -9.30 | -6.11 | 0.00 |
|  | 457-318 | -6.48 | -8.21 | -4.74 | 0.00 |
|  | 458-318 | -8.02 | -9.71 | -6.34 | 0.00 |
|  | 480-318 | -8.19 | -9.97 | -6.42 | 0.00 |
|  | 481-318 | -7.30 | -10.09 | -4.51 | 0.00 |
|  | 492-318 | -4.48 | -8.85 | -0.10 | 0.04 |
| LiDAR F5m | 318-289 | 4.66 | 1.23 | 8.09 | 0.00 |
|  | 318-290 | 2.89 | 0.49 | 5.30 | 0.00 |
|  | 318-312 | 5.25 | 3.65 | 6.84 | 0.00 |
|  | 318-313 | 4.38 | 0.56 | 8.20 | 0.01 |
|  | 318-314 | 3.90 | 0.89 | 6.92 | 0.00 |
|  | 318-317 | 7.62 | 5.88 | 9.37 | 0.00 |
|  | 319-318 | -5.67 | -8.54 | -2.79 | 0.00 |
|  | 320-318 | -7.52 | -8.86 | -6.19 | 0.00 |
|  | 457-318 | -6.51 | -7.97 | -5.06 | 0.00 |
|  | 458-318 | -8.00 | -9.41 | -6.59 | 0.00 |
|  | 480-318 | -8.25 | -9.73 | -6.76 | 0.00 |
|  | 481-318 | -7.48 | -9.82 | -5.15 | 0.00 |
|  | 492-318 | -4.21 | -7.87 | -0.54 | 0.01 |

**Table S10. ANOVA and Tukey’s HSD Test between Block 318 and other forest management blocks (continued)**

| Slope Raster | FMB Comparison | diff | lwr | upr | p adj |
| --- | --- | --- | --- | --- | --- |
| DTM | 318-289 | 4.75 | 0.57 | 8.93 | 0.01 |
|  | 318-290 | 3.65 | 0.72 | 6.57 | 0.00 |
|  | 318-312 | 7.17 | 5.22 | 9.11 | 0.00 |
|  | 318-313 | 5.37 | 0.71 | 10.02 | 0.01 |
|  | 318-314 | 5.76 | 2.10 | 9.43 | 0.00 |
|  | 318-317 | 9.94 | 7.82 | 12.07 | 0.00 |
|  | 319-318 | -7.19 | -10.69 | -3.69 | 0.00 |
|  | 320-318 | -8.74 | -10.37 | -7.11 | 0.00 |
|  | 457-318 | -7.18 | -8.95 | -5.41 | 0.00 |
|  | 458-318 | -8.82 | -10.54 | -7.10 | 0.00 |
|  | 480-318 | -8.48 | -10.29 | -6.68 | 0.00 |
|  | 481-318 | -7.88 | -10.73 | -5.04 | 0.00 |
|  | 492-318 | -5.87 | -10.34 | -1.41 | 0.00 |
| SRTM | 318-289 | 4.87 | 1.15 | 8.60 | 0.00 |
|  | 318-290 | 1.49 | -1.12 | 4.10 | 0.82 |
|  | 318-312 | 4.09 | 2.36 | 5.82 | 0.00 |
|  | 318-313 | 2.20 | -1.95 | 6.35 | 0.89 |
|  | 318-314 | 4.45 | 1.18 | 7.72 | 0.00 |
|  | 318-317 | 8.00 | 6.10 | 9.89 | 0.00 |
|  | 319-318 | -8.25 | -11.37 | -5.12 | 0.00 |
|  | 320-318 | -7.92 | -9.37 | -6.47 | 0.00 |
|  | 457-318 | -6.44 | -8.02 | -4.86 | 0.00 |
|  | 458-318 | -5.75 | -7.29 | -4.22 | 0.00 |
|  | 480-318 | -6.35 | -7.96 | -4.74 | 0.00 |
|  | 481-318 | -5.75 | -8.29 | -3.21 | 0.00 |
|  | 492-319 | 5.64 | 0.88 | 10.40 | 0.01 |
