# Supplementary material for: The use of spatial data and satellite information in legal compliance and planning in forest management
Source: PLoS One. 2022 Jul 27;17(7):e0267959. doi: 10.1371/journal.pone.0267959 (PMC9328540; doi:10.1371/journal.pone.0267959)
Supplement: S11 Table — (DOCX) [file pone.0267959.s016.docx]

**Table S11. Transects measured by the authors (ANU Transects)**

| Coupe address | Transect  reference | Length of Transect (m) | Measured Slope (degrees) | LiDAR Mean Slope (degrees) | VicMap DTM Mean Slope (degrees) | STRM Mean Slope (degrees) |
| --- | --- | --- | --- | --- | --- | --- |
| 318-512-0009 | TS1 | 24 | 29.6 | 27.56 | 44.33 | 26.47 |
|  | TS2 | 27.5 | 32 | 30.55 | 44.49 | 26.47 |
|  | TS3 | 25.5 | 32 | 31.46 | 42.83 | 19.90 |
|  | TS4 | 19.5 | 32 | 30.79 | 38.74 | 21.63 |
|  | TS5 | 22 | 35.2 | 32.23 | 31.63 | 34.38 |
|  | TS6 | 11.5 | 42.8 | 34.81 | 35.68 | 34.64 |
|  | TS7 | 10 | 39.4 | 35.35 | 34.86 | 32.13 |
|  | TS8 | 34.5 | 30.6 | 28.79 | 32.77 | 25.04 |
|  | TS9 | 22 | 31.2 | 29.99 | 35.64 | 25.71 |
|  | TS10 | 22 | 31 | 31.08 | 30.79 | 27.17 |
|  | TS11 | 33.5 | 31.6 | 30.69 | 31.36 | 27.58 |
| 318-512-0018 | TS12 | 12.5 | 34 | 31.79 | 28.80 | 35.57 |
|  | TS13 | 30 | 33.8 | 33.79 | 36.17 | 33.29 |
|  | TS14 | 32.5 | 31.6 | 31.23 | 35.16 | 28.94 |
|  | TS15 | 22 | 37.4 | 37.09 | 30.38 | 29.52 |
|  | TS16 | 27.5 | 36.8 | 35.68 | 33.06 | 29.45 |
|  | TS17 | 21.5 | 30 | 29.52 | 30.35 | 26.64 |
| 457-504-0006 | TS18 | 24.5 | 29 | 29.37 | 30.40 | 28.12 |
|  | TS19 | 31.5 | 30.6 | 29.24 | 27.59 | 23.11 |
|  | TS20 | 18.5 | 32 | 31.87 | 21.52 | 27.21 |
|  | TS21 | 29.5 | 33.6 | 32.18 | 32.01 | 25.32 |
|  | TS22 | 26 | 33.8 | 33.43 | 30.53 | 28.82 |
|  | TS23 | 22 | 33 | 29.18 | 32.41 | 25.83 |
|  | TS24 | 23.5 | 31.6 | 29.59 | 30.78 | 29.47 |
|  | TS25 | 16.5 | 35 | 30.59 | 29.00 | 26.27 |
|  | TS26 | 12 | 34 | 30.15 | 26.65 | 26.80 |
