# Supplementary material for: The use of spatial data and satellite information in legal compliance and planning in forest management
Source: PLoS One. 2022 Jul 27;17(7):e0267959. doi: 10.1371/journal.pone.0267959 (PMC9328540; doi:10.1371/journal.pone.0267959)
Supplement: S12 Table — (DOCX) [file pone.0267959.s017.docx]

**Table S12. Transects measured by the Timber Harvest Compliance Unit of the Office of the OCR (OCR Transects)**

| Coupe address | Transect  reference | Length of Transect (m) | Measured Slope (degrees) | LiDAR Mean Slope (degrees) | VicMap DTM Mean Slope (degrees) | STRM Mean Slope (degrees) |
| --- | --- | --- | --- | --- | --- | --- |
| 318-512-0010 | TS1 | 55.02 | 28.4 | 29.23 | 33.38 | 23.31 |
|  | TS2 | 57.63 | 30.6 | 30.28 | 34.15 | 26.95 |
|  | TS3 | 50.05 | 31.6 | 32.84 | 38.06 | 30.93 |
|  | TS4 | 84.61 | 31.4 | 33.14 | 39.02 | 25.49 |
|  | TS5 | 53.59 | 33.2 | 33.07 | 40.27 | 28.85 |
|  | TS6 | 102.61 | 31.2 | 33.85 | 35.74 | 27.09 |
|  | TS7 | 63.25 | 31.2 | 30.44 | 37.27 | 32.74 |
|  | TS8 | 81.45 | 31.2 | 33.87 | 32.98 | 28.24 |
|  | TS9 | 58.37 | 29.6 | 30.77 | 33.99 | 27.32 |
|  | TS10 | 64.81 | 26.8 | 27.22 | 32.63 | 29.89 |
|  | TS11 | 62.27 | 27.8 | 28.30 | 32.46 | 31.06 |
|  | TS12 | 51.44 | 30.2 | 30.37 | 33.22 | 26.50 |
|  | TS13 | 51.86 | 32.0 | 31.53 | 38.43 | 29.47 |
|  | TS14 | 30.51 | 32.0 | 32.07 | 42.39 | 29.34 |
|  | TS15 | 31.92 | 30.9 | 30.81 | 39.26 | 25.21 |
| 320-501-0024 | TS16 | 25.56 | 28.2 | 30.15 | 15.68 | 28.82 |
|  | TS17 | 38.05 | 28.6 | 30.57 | 18.43 | 28.37 |
|  | TS18 | 52.71 | 27.4 | 28.50 | 20.33 | 26.80 |
|  | TS19 | 52.08 | 29.0 | 29.79 | 25.16 | 24.72 |
|  | TS20 | 42.55 | 30.0 | 31.25 | 22.36 | 26.58 |
|  | TS21 | 35.91 | 30.8 | 34.30 | 22.21 | 24.92 |
|  | TS22 | 31.09 | 32.0 | 32.95 | 26.82 | 26.00 |
|  | TS23 | 30.89 | 38.0 | 33.77 | 24.72 | 28.90 |
|  | TS24 | 40.35 | 33.0 | 34.32 | 23.44 | 36.33 |
|  | TS25 | 52.87 | 33.3 | 32.89 | 28.45 | 31.41 |
|  | TS26 | 43.34 | 28.8 | 28.88 | 39.59 | 24.87 |
|  | TS27 | 58.12 | 30.4 | 31.63 | 37.93 | 29.51 |
|  | TS28 | 52.59 | 32.4 | 33.31 | 39.25 | 25.64 |
|  | TS29 | 21.62 | 34.0 | 34.17 | 32.00 | 28.48 |
|  | TS30 | 26.92 | 31.2 | 31.47 | 26.51 | 21.67 |
|  | TS31 | 53.31 | 32.8 | 25.70 | 15.63 | 22.18 |
|  | TS32 | 31.32 | 24.5 | 25.55 | 24.87 | 22.65 |
|  | TS33 | 14.89 | 30.9 | 31.41 | 28.35 | 26.60 |
|  | TS34 | 79.62 | 22.0 | 24.25 | 38.82 | 28.00 |
|  | TS35 | 36.26 | 29.6 | 31.19 | 36.43 | 26.74 |
|  | TS36 | 21.59 | 27.0 | 28.04 | 36.32 | 28.43 |
