# Supplementary material for: The use of spatial data and satellite information in legal compliance and planning in forest management
Source: PLoS One. 2022 Jul 27;17(7):e0267959. doi: 10.1371/journal.pone.0267959 (PMC9328540; doi:10.1371/journal.pone.0267959)
Supplement: S13 Table — Adjusted R2 Values were generated from an Ordinary Least Squares test. (DOCX) [file pone.0267959.s018.docx]

**Table S13. Wilcoxon signed rank test comparing measured slope to the average slope calculations derived from the LiDAR 1m DEM, the VicMap Elevation DTM and the SRTM DEM. Adjusted R^2^ Values were generated from an Ordinary Least Squares test**

| **Transect** | **Comparison** | **V** | **P-Value** | **Adjusted R^2^** |
| --- | --- | --- | --- | --- |
| ANU | LiDAR 1m-Measured Slope | 342 | 9.835e-07 | 0.6405 |
|  | DTM-Measured Slope | 204 | 0.4834 | -0.04033 |
|  | SRTM-Measured Slope | 347 | 2.086e-07 | 0.3007 |
| OCR | LiDAR 1m-Measured Slope | 120 | 0.0005099 | 0.5867 |
|  | DTM-Measured Slope | 286 | 0.4697 | -0.02941 |
|  | SRTM-Measured Slope | 571 | 7.807e-05 | 0.008964 |
