# Supplementary material for: The use of spatial data and satellite information in legal compliance and planning in forest management
Source: PLoS One. 2022 Jul 27;17(7):e0267959. doi: 10.1371/journal.pone.0267959 (PMC9328540; doi:10.1371/journal.pone.0267959)
Supplement: S14 Table — (DOCX) [file pone.0267959.s019.docx]

**Table S14. Descriptive statistics of the ANU and OCR Transects covering measured slope and the average slope calculations generated from the LiDAR 1m DEM, the VicMap Elevation DTM and the SRTM DEM**

| Transects | Data (Slope in Degrees) | Min | 1st Qu | Median | Mean | 3rd Qu | Max | Sd |
| --- | --- | --- | --- | --- | --- | --- | --- | --- |
| ANU | Measured | 29.00 | 31.30 | 32.00 | 33.22 | 34.00 | 42.80 | 3.14 |
|  | LiDAR 1m | 27.56 | 29.69 | 30.93 | 31.46 | 32.22 | 37.09 | 2.34 |
|  | DTM | 21.52 | 30.39 | 31.82 | 33.00 | 35.52 | 44.49 | 5.29 |
|  | SRTM | 19.90 | 25.94 | 27.19 | 27.90 | 29.47 | 35.57 | 3.81 |
| ANU | LiDAR 1m - Measured | -7.99 | -2.16 | -1.29 | -1.75 | -0.40 | 0.37 | 1.86 |
|  | DTM - Measured | -10.48 | -4.34 | -0.70 | -0.22 | 2.32 | 14.73 | 6.25 |
|  | SRTM - Measured | -12.10 | -7.45 | -5.51 | -5.31 | -3.19 | 1.57 | 3.27 |
| OCR | Measured | 22.00 | 28.75 | 30.85 | 30.33 | 32.00 | 38.00 | 2.83 |
|  | LiDAR 1m | 24.25 | 29.65 | 31.22 | 30.89 | 32.98 | 34.32 | 2.59 |
|  | DTM Slope | 15.63 | 25.09 | 33.10 | 31.29 | 37.96 | 42.39 | 7.51 |
|  | SRTM Slope | 21.67 | 25.60 | 27.21 | 27.50 | 29.01 | 36.33 | 3.00 |
| OCR | LiDAR 1m - Measured | -7.10 | 0.08 | 0.87 | 0.55 | 1.26 | 3.50 | 1.84 |
|  | DTM - Measured | -17.17 | -4.93 | 3.97 | 0.96 | 6.84 | 16.82 | 8.03 |
|  | SRTM - Measured | -10.62 | -5.20 | -3.19 | -2.83 | -0.65 | 6.00 | 3.71 |
