# Supplementary material for: The use of spatial data and satellite information in legal compliance and planning in forest management
Source: PLoS One. 2022 Jul 27;17(7):e0267959. doi: 10.1371/journal.pone.0267959 (PMC9328540; doi:10.1371/journal.pone.0267959)
Supplement: S15 Table — (DOCX) [file pone.0267959.s020.docx]

**Table S15. Cut blocks calculated with slopes >30° logged across the Upper Goulburn water supply protection area as calculated by VicForests using a resampled LiDAR DEM at 10m resolution.**

| Catchment | Metric | Cut Block Category | LiDAR 10m VicForests | LiDAR F5m (this study) |
| --- | --- | --- | --- | --- |
| Upper Goulburn | No. of Cut Blocks | All Slopes <30° | 63 | 32 |
|  |  | 0-1% Area >30° | 54 | 53 |
|  |  | 1-5% Area >30° | 34 | 54 |
|  |  | 5-10% Area >30° | 13 | 18 |
|  |  | >10% Area >30° | 10 | 15 |
|  |  | Sub Total Cut blocks >30° | 111 | 140 |
| Upper Goulburn | Area logged >30° | Subtotal (ha) | 80.7 | 79.1 |
|  |  | % of Total Area Logged | 2.6% | 3.1% |
